# A spontaneous bilateral fallopian tube pregnancy with didelphic uterus: A case report

**DOI:** 10.1097/MD.0000000000024291

**Published:** 2021-01-15

**Authors:** Xi Zeng, Li Luo, Yun-Wei Ou-Yang

**Affiliations:** Department of Gynecology and Obstetrics, The West China Second University Hospital, Sichuan University, Key Laboratory of Birth Defects and Related Diseases of Women and Children (Sichuan University), Ministry of Education, Chengdu, China.

**Keywords:** bilateral fallopian tube pregnancies, didelphic uterus, pathology, surgery

## Abstract

**Rationale:**

In this article, we report interesting clinical manifestation of spontaneous bilateral fallopian tube pregnancies in a patient with a didelphic uterus.

**Patient concerns:**

A 26-year-old female patient, gravida 2, para 0 + 1, suffered from progressive abdominal pain and vaginal bleeding. A laboratory exam revealed a human chorionic gonadotropin level of 1091 IU/L. Transvaginal ultrasound detected no embryo sacs in the uterus but revealed a didelphic uterus, and a mass measuring 39 mm x 32 mm in the left adnexa region with another mass measuring 42 x 28 mm in the right adnexa region.

**Diagnoses:**

An ectopic pregnancy in the left adnexa region and a corpus hemorrhagicum in the right adnexa region were suspected.

**Interventions:**

Laparoscopic exploration operation confirmed a didelphic uterus, and pathological biopsy revealed bilateral fallopian tube pregnancies.

**Outcomes:**

The patient made a good recovery and the human chorionic gonadotropin became normal within the following 2 months.

**Lessons:**

To the best of our knowledge, clinical manifestation of spontaneous bilateral fallopian tube pregnancies in a patient with a didelphic uterus has never been reported before. Based on the experience with this case, we suggest that if a gestational sac is found in 1 fallopian tube, the contralateral fallopian tube needs to be examined for an ectopic pregnancy during surgery.

## 1 Introduction

Ectopic pregnancy (EP) is a common emergency in gynecology. EP accounts for 1% to 2% of all pregnancies and is the most common cause of first trimester maternal death.^[1]^ More than 98% of EPs involve implantation in the fallopian tube, and bilateral ectopic pregnancies of the fallopian tubes are estimated to account for 1 in 750 to 1 in 1580 ectopic pregnancies.^[2,3]^

During the 8th to 12th weeks of embryonic development, the caudal Mullerian ducts fuse to become the uterus. Partial or complete failure of the Mullerian ducts to fuse leads to the formation of a didelphic uterus, which is characterized by 2 uterine bodies and/or 2 cervices.^[4]^

We herein present a case of bilateral fallopian tube pregnancies in a patient with a didelphic uterus.

## 2 Case presentation

A 26-year-old woman (gravida 2, para 0 + 1) who conceived naturally was admitted to our hospital with progressive abdominal pain and mild vaginal bleeding of 4 days duration. There was no history of surgery or other disease. Additionally, the patient denied any family disease history or drug history, including the use of ovulation induction drugs or combined oral contraceptives. Her previous pregnancy was spontaneously aborted at a gestational age of approximately 4 weeks, 2 years before admission.

On physical examination, her heart rate was 100 beats/min, and her blood pressure was 111/85 mm Hg. Her abdomen was tender with slight acute left adnexal pain, and only 1 cervix was noticed. The remaining physical examination, including the right adnexal region, did not reveal any additional abnormalities.

**Figure 1:**
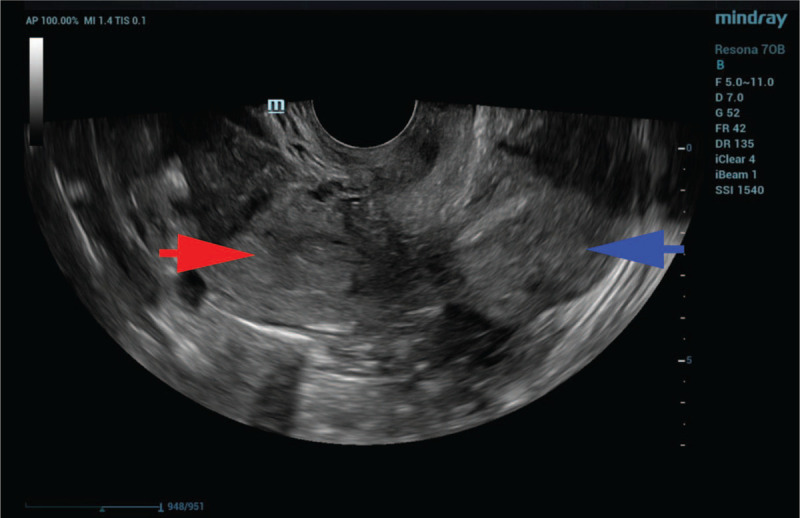
Transvaginal ultrasound before surgery confirmed a didelphic uterus. The red arrow indicates the right uterus. The blue arrow indicates the left uterus.

**Figure 2:**
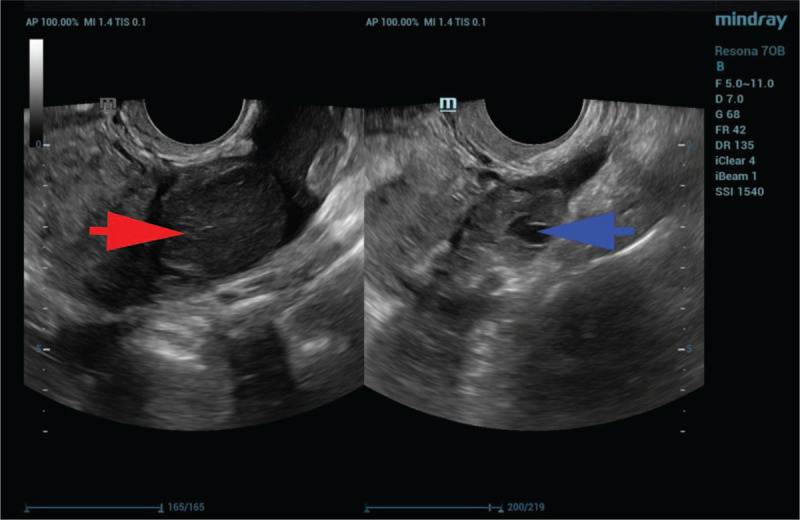
Transvaginal ultrasound before surgery confirmed masses in the bilateral adnexa regions. The red arrow indicates an EP in the right adnexa region. The blue arrow indicates the left adnexa region.

On laboratory examination, the human chorionic gonadotropin level was 786.3 IU/L, and 2 days later, it was 1091 IU/L. Her hemoglobin was 116 g/L. The remaining laboratory results were unremarkable. Transvaginal ultrasound did not detect embryo sacs in the uterus but revealed a didelphic uterus (Fig. 1), a mass measuring 39 mm x 32 mm in the left adnexa region and a mass measuring 42 x 28 mm in the right adnexa region (Fig. 2). A total of 3.1 cm of Pelvic effusion was also present in Douglas’ pouch. With a suspected EP in the left adnexa region and a corpus hemorrhagicum in the right adnexa region, laparoscopy surgery was scheduled. The estimated gestational age was approximately 6 weeks, given her last menstrual period.

**Figure 3:**
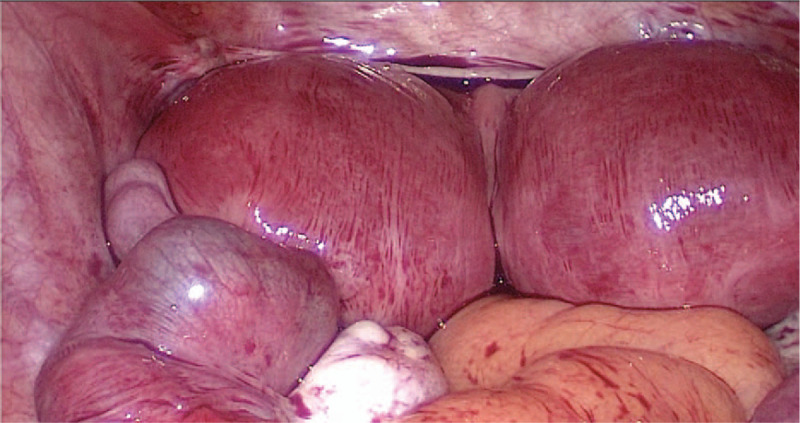
On laparoscopy, the didelphic uterus was present and 12 cm× 9 cm× 7 cm in size, and ectopic pregnancy embryo sacs were present in the left fallopian tube and 4 cm× 3 cm in size.

**Figure 4:**
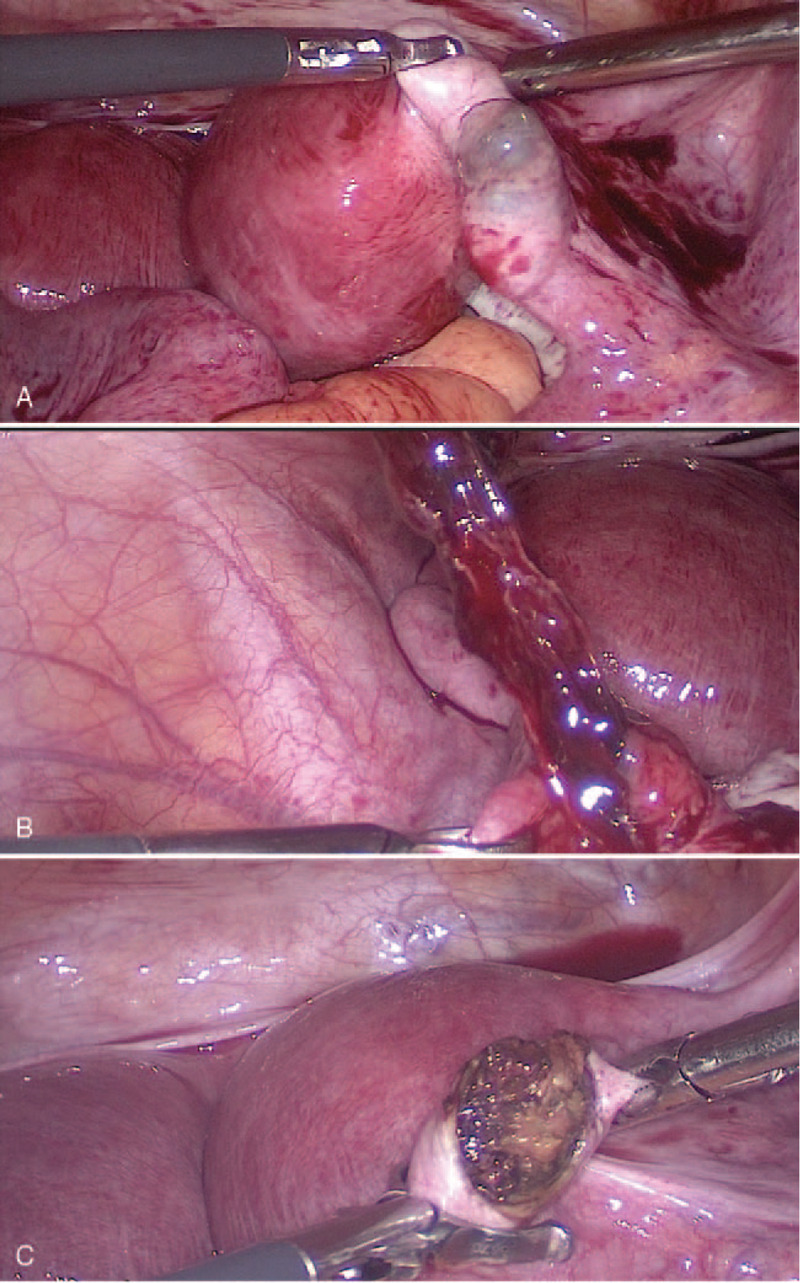
A. Another ectopic pregnancy embryo sac in the right fallopian tube, approximately 3 cm× 2 cm in size. B and C. The embryo sacs in the left (B) and right (C) fallopian tubes were removed during the surgery.

**Figure 5:**
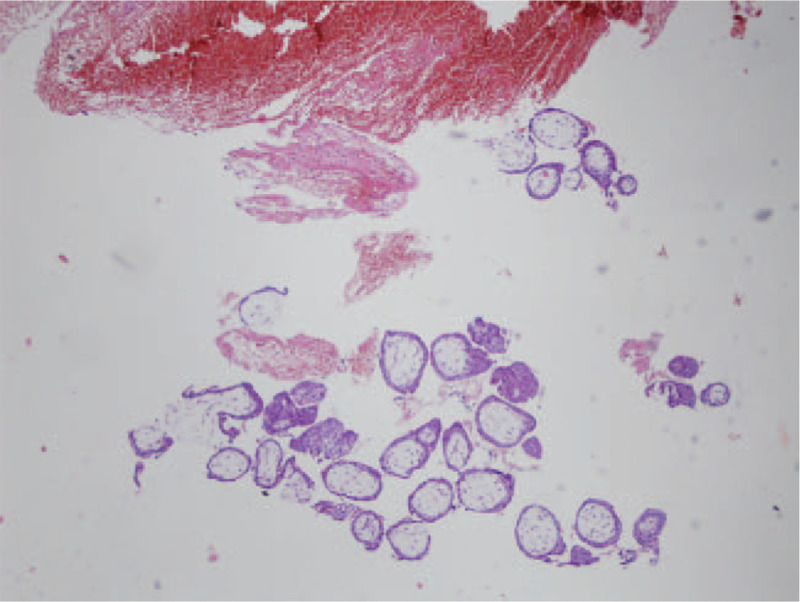
Pathology examinations confirmed placental villus tissues.

During the explorative laparotomy, approximately 100 mL of hemoperitoneum was drained. A didelphic uterus was present, with a size of 12 cm× 9 cm× 7 cm (Fig. 3). The bilateral fallopian tubes were edematous and dilated. Simultaneously, there was a definite left EP (Fig. 4-A) and a possible hematosalpinx or EP on the right (Fig. 4-B). As a result, laparoscopic removal of the bilateral fallopian tube lesions was performed (Fig. 4-C), and the total blood loss was estimated to be 70 mL. All tissue samples were sent for pathology. The pathology specimen confirmed the twin tubal pregnancies (Fig. 5).

**Figure 6:**
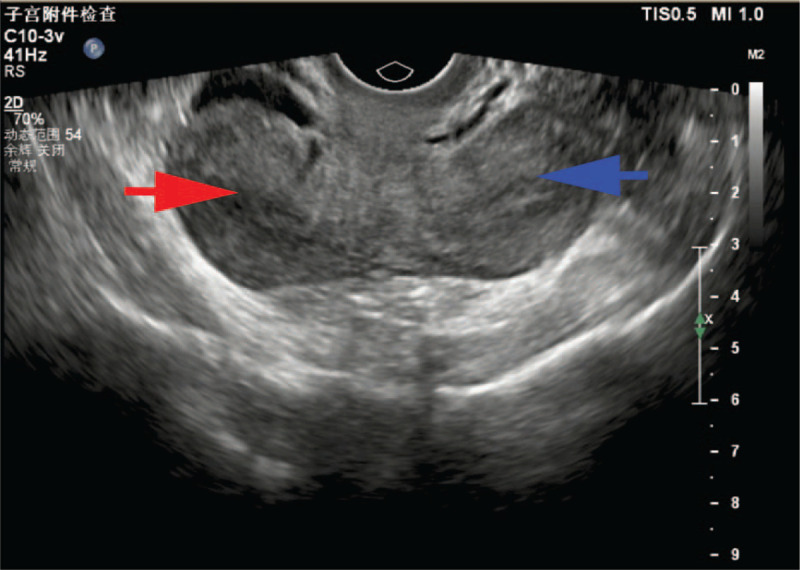
Transvaginal ultrasound postsurgery confirmed a didelphic uterus with no mass in the bilateral adnexa regions. The red arrow indicates the right uterus. The blue arrow indicates the left uterus.

The postoperative course was regular, and the patient was discharged home 2 days later; she returned for follow-up 2 weeks later. The ultrasound scan was negative, and the human chorionic gonadotropin level began to decrease, returning to normal 2 months later. Transvaginal ultrasound confirmed a didelphic uterus with no mass in the bilateral adnexa regions (Fig. 6).

## 3 Discussion

In 1986, Santos first reported a case of unruptured twin tubal pregnancy.^[5]^ Although in recent years, due to the development of assisted reproductive technology, the morbidity of EP has increased, spontaneous bilateral fallopian tube pregnancy cases remain rare.^[6]^ Transvaginal ultrasound has a positive predictive value of 96.7% and a negative predictive value of 99.4% for the identification of EP.^[6–8]^ However, the efficacy of preoperative ultrasound in diagnosing bilateral EPs is also poor, with only a couple of successful cases known because even the most experienced sonographers may only encounter a handful, and no previous literature has reported cases.^[9]^

The incidence of didelphic uterus is approximately 10% of all uterine anomalies.^[10]^ The endosalpinx produced by sexually transmitted infections, distortion of pelvic anatomy caused by endometriosis or adhesions after a pelvic operation and congenital anomalies can be risk factors for EP.^[11]^ Additionally, previous history of EP, tubal surgery or ligation increase the possibility of an EP.^[12]^

Our case is particularly peculiar on 2 counts. First, it is a spontaneous bilateral fallopian tube pregnancy; similar cases do not exist.^[6]^ Second and more importantly, the patient had a didelphic uterus. The diagnosis of EP in this patient was further concealed by her unremarkable medical history and physical examination findings. Based on our literature review, this is the first case report of bilateral fallopian tube pregnancies in a patient with a didelphic uterus.

## 4 Conclusion

The article reiterates that when a pregnant patient presents with first-trimester bleeding or abdominal pain, physicians should consider EP as a possible cause, regardless of whether her uterus has congenital anomalies. More importantly, even if a gestational sac is found in 1 fallopian tube, the contralateral fallopian tube needs to be examined for an EP during surgery.

## Author contributions

Luo Li: Critical revision of article.

Ou-Yang Yun-Wei: Design, Approval of article.

Zeng Xi: Case collection, Drafting article.

**Conceptualization:** Yun-Wei Ou-Yang.

**Data curation:** Xi Zeng.

**Supervision:** Li Luo, Yun-Wei Ou-Yang.

**Writing – original draft:** Xi Zeng.

**Writing – review & editing:** Li Luo, Yun-Wei Ou-Yang.

## Abbreviations

Abbreviation: EP = ectopic pregnancy.
